# Prevalence and adverse outcomes of twin pregnancy in Eastern Africa: a systematic review and meta-analysis

**DOI:** 10.1186/s12884-024-06326-0

**Published:** 2024-02-29

**Authors:** Tamirat Getachew, Abraham Negash, Adera Debella, Elias Yadeta, Magersa Lemi, Bikila Balis, Tegenu Balcha, Habtamu Bekele, Mohammed Abdurke, Addisu Alemu, Kasiye Shiferaw, Addis Eyeberu

**Affiliations:** 1 School of Nursing and Midwifery, College of Health and Medical Sciences, Haramaya University, P.O. BOX 138, Dire Dawa, Harar, Ethiopia; 2 School of Public Health, College of Health and Medical Sciences, Haramaya University, Harar, Ethiopia

**Keywords:** Twin pregnancy, Multiple pregnancies, Pregnancy outcome, Meta-analysis

## Abstract

**Introduction:**

Multiple pregnancies are much more common today than they were in the past. Twin pregnancies occur in about 4% of pregnancies in Africa. Adverse pregnancy outcome was more common in twin pregnancy than in singleton pregnancy. There is no pooled evidence on the burden and adverse pregnancy outcome of twin pregnancy in eastern Africa. Thus, this systematic review and meta-analysis were conducted to assess the prevalence and adverse pregnancy outcomes of twin pregnancies.

**Methods:**

This systematic review and meta-analysis covers published and unpublished studies searched from different databases (PubMed, CINAHL (EBSCO), EMBASE, DOAJ, Web of Sciences, MEDLINE, Cochrane Library, SCOPUS, Google Scholar, and Google search). Finally, 34 studies were included in this systematic review and meta-analysis. JBI checklist was used to assess the quality of included papers. Preferred Reporting Items for Systematic Reviews and Meta-Analyses (PRISMA) guidelines were used. Data synthesis and statistical analysis were conducted using STATA Version 14 software. Heterogeneity and publication bias were assessed. A forest plot was used to present the pooled prevalence using the random effect model.

**Results:**

The prevalence of twin pregnancy in eastern Africa was 3% [95% CI: 2, 3]. The adverse pregnancy outcomes like neonatal intensive care unit admission (78%), low birth weight (44%), low APGAR score (33%), prematurity (32%), stillbirth (30%), neonatal mortality (12%) and maternal complications like hypertensive disorder of pregnancy (25%), postpartum hemorrhage (7%), Cesarean section (37%), premature rupture of membrane (12%) and maternal mortality are more common among twin pregnancy than singleton pregnancy.

**Conclusion:**

One in every 33 children born a twin in east Africa; admission to neonatal intensive care unit, low birth weight, low APGAR score, prematurity, stillbirth, neonatal mortality and maternal complications are its associated adverse birth outcomes. Since twin pregnancy is a high-risk pregnancy, special care is needed during pregnancy, labor and delivery to reduce adverse pregnancy outcomes.

**Supplementary Information:**

The online version contains supplementary material available at 10.1186/s12884-024-06326-0.

## Introduction

Globally about 3 million neonatal death occurs yearly [1]. Almost all (99%) of these deaths occur in low-income countries with inadequate facilities [2]. Twin pregnancy increases the risk adverse pregnancy outcomes such as stillbirth, preterm birth, postpartum hemorrhage and maternal mortality [3–5].

Twin pregnancy occurs in about 0.6% of all pregnancies in Asia, 1-2 % in Australia, Europe, and the United States of America, and about 4% in Africa [6, 7].The incidence of multiple pregnancies has increased by 50% since 1980 [8–10], making them more common today than in the past. About 50% of twin pregnancies result from assisted reproductive technology, a treatment of infertility [11, 12].

Multiple pregnancy is associated with adverse prenatal outcomes, as the singleton risk is multiplied by the number of fetuses [9]. It accounts for 12.5% of prenatal mortality [7]. Data from 30 nations in Sub-Saharan Africa revealed that twin pregnancy has a five-fold greater infant mortality rate than singleton pregnancy [13, 14].

The number of perinatal complications rises with multiple gestations. Twin pregnancies are linked with a higher risk of unfavorable perinatal outcomes, including fetal anomalies, prenatal morbidity and mortality [15, 16], preterm birth and intrauterine growth restriction [17], low APGAR scores, low birth weight, early neonatal death, and admission to the NICU [3, 18].

Similarly, severe maternal morbidity, such as preeclampsia, and gestational diabetes [16, 19], as well as cesarean section, and induction of labour [19] maternal near miss and maternal death were more common in twin pregnancies than in singleton pregnancies [3, 20, 21].

Understanding the risks of a twin pregnancy before conception can aid in making decisions regarding fertility treatment [22]. Since multiple pregnancies pose a higher risk of mortality and morbidity for both mother and newborn compared to singleton pregnancies, it is advisable to seek essential and additional elements of care from multidisciplinary teams [23, 24].

Understanding the pooled prevalence and prenatal outcome of twin pregnancies is crucial for developing a care plan that ensure optimal and timely delivery. This is a key strategy for reducing perinatal morbidity and mortality associated with twin pregnancies [25, 26]. Despite this importance there is currently a lack of summary of evidence about the burden of twin pregnancy and its consequences in Eastern Africa. Therefore, this study aimes to determine the pooled prevalence of twin pregnancy and its adverse pregnancy outcomes in the region.

## Methods

### Protocol and registration

This review was aimed to identify the pooled prevalence of twin pregnancy and adverse outcomes in Eastern Africa following the Preferred Reporting Items for Systematic Reviews and Meta-Analyses (PRISMA) guideline [27] (Additional file 1). It was registered by the International Prospective Register of Systematic Reviews (PROSPERO), ID: CRD42022338393.

### Eligibility criteria

Including studies conducted in Eastern Africa that assessed the prevalence and/or outcome of twin pregnancies. Observational studies (cross-sectional, cohort, and case-control) reported outcomes of interest in eastern Africa were included. This review included articles conducted in Eastern Africa and published until 03 June 202, with all full-text articles written in English. Experimental studies, reviews, commentaries, editorials, and case series/reports were not included in this review.

### Data sources and search strategy

Articles for this systematic review and meta-analysis were retrieved through electronic web-based searches on multiple data base including PubMed, EMBASE, CINHAL (EBSCO), POPLINE, Google Scholar, DOAJ, Web of Sciences, MEDLINE, Cochrane Library, SCOPUS, Google search, and Mednar. These searches employed a combination of Boolean logic operators (AND, OR, NOT), Medical Subject Headings (MeSH), and keywords.

The search strategy for advanced PubMed includes ("pregnancy, twin"[MeSH Terms] OR ("pregnancy, twin"[MeSH Terms] OR ("pregnancy"[All Fields] AND "twin"[All Fields]) OR "twin pregnancy"[All Fields] OR ("twin"[All Fields] AND "pregnancies"[All Fields]) OR "twin pregnancies"[All Fields]) OR ("pregnancy, twin"[MeSH Terms] OR ("pregnancy"[All Fields] AND "twin"[All Fields]) OR "twin pregnancy"[All Fields] OR ("twins"[All Fields] AND "pregnancy"[All Fields]) OR "twins pregnancy"[All Fields]) OR ("pregnancy, multiple"[MeSH Terms] OR ("pregnancy"[All Fields] AND "multiple"[All Fields]) OR "multiple pregnancy"[All Fields] OR ("multiple"[All Fields] AND "gestation"[All Fields]) OR "multiple gestation"[All Fields]) OR "pregnancy, multiple"[MeSH Terms]) AND ("perinatal outcome"[All Fields] OR "adverse outcome"[All Fields] OR "pregnancy outcome"[MeSH Subheading] OR "maternal outcome"[All Fields] OR "fetal outcome"[All Fields] AND "africa, eastern"[MeSH Terms].

For Scopus search: In addition the search strategy “Twin pregnancy OR Twins pregnancy OR Multiple pregnancy OR Twin pregnancies OR Multiple gestation AND (perinatal OR maternal OR fetal outcomes) AND Eastern Africa” was used considering fields, and title/abstract. The search was extended to include the above search terms in each of the following countries independently: Burundi, Comoros, Djibouti, Ethiopia, Eritrea, Kenya, Rwanda, Seychelles, Somalia, South Sudan, Sudan, Tanzania, and Uganda. The detailed search strategy for all databases is outlined in (Additional file 2). All identified keywords and index terms were checked across all databases. Finally, the reference lists of all identified articles were searched for further relevant articles.

### Study selection

**Fig. 1:**
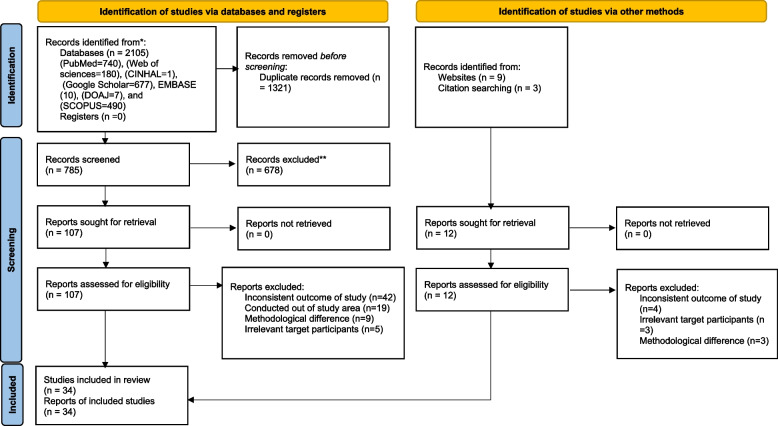
PRISMA flow diagram of studies included in final systematic review and meta-analysis of the prevalence of twin pregnancy in eastern Africa

The reference management software (Endnote version X8) was primarily used to combine database search results and manually remove duplicate articles. Titles and abstracts were thoroughly evaluated and the full text of the remaining articles was reviewed for eligibility by five independent authors (TG, AN, AE, MA, and BB) based on predetermined inclusion and exclusion criteria. Full-text articles in English were further evaluated based on objectives, methods, population, and key findings (Prevalence/Magnitude, outcomes of twin pregnancy, and Eastern Africa). Any uncertainties during the extraction process were resolved through logical consensus among the five authors, and the final consensus was approved with the participation of authors (HB and ML). The overall study selection process is presented using the PRISMA statement flow diagram (Fig. 1).

### Data extraction

The authors (TG, AN, AE, MA, and BB) independently extracted the data from the retrieved papers. The information from included studies was entered into a pre-made Microsoft Excel 2016 sheet with the following headings: author and year of publication, country, study setting, study design, study subject, data collection methods, sample size, number of cases, prevalence of twin pregnancy, and maternal and neonatal outcomes. To ensure accuracy three researchers (AD, HB, and MA) independently extracted the data from 30% of the included article to verify the correctness of the data extraction.

### Data Item

The outcome variables of interest included prevalence and adverse maternal and neonatal outcomes of twin pregnancies. The maternal outcomes encompassed maternal death (death before seven days postpartum or discharge), and severe adverse maternal outcome index was also used (maternal death, postpartum hemorrhage, hypertensive disorders, premature rupture of membrane, cesarean section). The perinatal outcomes consisted of stillbirth (an infant born with no signs of life), early neonatal death (live-born neonate that died in the first seven days of life, before discharge), perinatal mortality (stillbirth and early neonatal death), congenital anomalies, low Apgar score, admission to NICU, and preterm delivery. This review included all studies that used the above-mentioned definitions.

### Risk of bias

Investigators critically evaluated the risk of bias in individual studies using the Joanna Briggs Institute Quality Assessment Tool for observational studies [28]. To minimize bias comprehensive searches (electronic/database search and manual search) were conducted, including published and unpublished, institutional, or community-based studies. The collaborative efforts of authors played a crucial role in reducing bias, by establishing a clear schedule for the selection of articles based on predefined objectives and eligibility criteria, determining article quality regularly evaluating the review process, and extracting and compiling the data.

### Critical appraisal of studies

The methodological reputability and quality of included studies were critically assessed using the Joanna Briggs Institute (JBI) quality assessment tool for observational studies (cross-sectional, case-control, and cohort studies) [28] (Additional file 3). The two group authors (TG and AN) and (AE, BB, and MA) independently evaluated the quality of the studies. The mean score of the two groups was considered for final decision and any discrepancies in study inclusion were resolved through consensus. The included studies were evaluated against each indicator of the tool and categorized as high, moderate, and low quality with high-quality scoring above 80%, moderate quality between 60%-80%, and low quality below 60%. Studies with a score greater than or equal to 60% were included in systematic review and meta-analysis. The critical appraisal aimed to assess both the internal validity (systematic error) and external validity (generalizability) of studies thereby reducing the risk of biases.

### Statistical analysis

Data synthesis and statistical analysis were conducted using STATA 14 software. The meta-analysis results, illustrating the prevalence of twin pregnancies in eastern Africa, were presented using forest plots. The random effect model was employed to analyze the data and mitigate heterogeneity among included studies. Subgroup analyses were also conducted by different study characteristics such as publication year and study setting or countries. Furthermore, meta-analysis regression was conducted to identify the sources of heterogeneity among studies.

Following Higgins et al recommendations meta-analysis of observational studies was conducted considering an I^2^ statistic of 75/100% and above as an indicative of high heterogeneity. Publication bias was checked by visual inspection of a funnel plot and Egger’s Regression Test with *P*-values less than 0.10 indicating presence of publication bias. The review's findings were presented in accordance with PRISMA recommendations. A narrative synthesis, followed by a meta-analysis chart, was used to present the findings of the studies.

## Results

### Study selection

A total of 2118 articles were retrieved with 740 from PubMed, 490 from Scopus, 180 from Web of Science, 1 from CINAHL, 7 from DOAJ, 677 from Google Scholar, and 12 from other sources. From the total identified studies, 1321 articles were removed due to duplication using ENDNOTE and visual assessment. From the remaining 785 studies, 678 articles were excluded after screening the respective titles and abstracts. The eligibility of the remaining 119 full-text articles were assessed leading to exclusion of 85 studies that did not present the outcome of interest, were conducted outside of the study area and had methodological differences. Finally, 34 studies were included in this systematic review and meta-analysis (Fig. 1).

### Description of included studies

A total of 34 studies assessing the prevalence of twin pregnancies and/or adverse maternal and fetal outcomes in eastern Africa were included in this systematic review and meta-analysis. The included studies vary in sample size ranging from 719 in a cross-sectional study conducted in Tanzania [29] to 44605 in a cohort study done in Sudan [30]. Overall, this study included a total of 121,272 pregnant mothers, 155,436 postnatal mothers, and 32,693 neonates. The systematic review and meta-analysis included studies from Rwanda [2], Uganda [3], Burundi [3], Comoros [3], Sudan [5], Kenya [4], Ethiopia [6], and Tanzania [8], all located in eastern Africa. Most of the studies 28 (82.4%) utilized cross-sectional study design whereas the remained used cohort [4] and case control study design [2]. Chart review was the main methods of data collection among included studies. Moreover, 14 (41.2%) of the studies included in this systematic review and meta-analysis were facility-based, while the rest were analyses of demographic health surveys from various countries. All studies reported the prevalence of twin pregnancies. Some studies include both maternal and neonatal complications, whereas others only include either of the two (Table 1).

**Table 1:** Description of study participants and characteristics of studies included in the systematic review and meta-analysis

**Author and years of publication**	**Country**	**Study Setting**	**Study Design**	**Study subject**	**Data collection method**	**Sample size**	**No of Cases**	**Prevalence of twin pregnancy**	**Maternal and neonatal Outcome (prevalence)**
Kheir, A.E.M., R. Ali, and S.M.H. Abdelmonim, 2016 [31]	Sudan	Facility based	Cross-sectional	Pregnant Mothers	Interview and Observation	1600	200	1.25	**Neonatal:** NICU admission (81.3%),LBW (60.4%), Prematurity (66%), APGAR <7 at 5th min (37.4%), Early neonatal mortality (19.6%)
Bekabil, T.T., et al; 2015 [32]	Ethiopia	Facility based	Prospective Cohort	Pregnant Mothers	Interview	3668	144	3.9	**Neonatal**: LBW (I5.7%), Prematurity (37.8%), Low APGAR (54%), NICU admission (75%), Neonatal death (4.7%), mal-presentation (37.4%), Congenital malformation 2(0.7%) **Maternal: medical complications**: HTN (25%); Hyperemesis (8.0%), Diabetes (0.7% ) **Obstetric complications**: PROM (15.3%), poor progress of labor (8.3%), cord prolapse (4.9%), and polyhydramnos (1.4%), PPH (3.2%), puerperal sepsis (3.1%), and maternal death (2.8%)
Marete, Irene, et al., 2014 [33]	Kenya	Facility based	cross-sectional	pregnant mothers	Chart review	8953	131	1.4	**Neonatal**: Stillbirths (78.1%), Early Neonatal mortality (11.3%)
Abdul, M.A., 2000 [34]	Comoros	Facility based	cross-sectional	pregnant mothers	Chart review	4370	109	2.5	**Neonatal:** LBW (50%), Perinatal mortality (5.5%) Retained second twin (12%) **Maternal**: Uterine atony (99%)
Ayza, A., 2018 [35]	Ethiopia	Facility based	Cross-sectional	Postnatal mother	Chart review	4328	124	2.9	**Maternal**: HDP (23.1%), PROM (13.5%), Preterm (7.1%), C/S (44.2%), Anemia (12.2%), **Neonatal**: Cord prolapse (3.2%)
Elshibly EM and Schmalisch G, 2010, 2010 [36]	Sudan	Facility-based	Comparative cross sectional	Pregnant Mother	Interview and anthropometric measurement	1030	30	2.9	**Neonatal:** 66.7% of Twin A weigh greater than Twin B.
Musili, F. and J. Karagja, 2009 [37]	Sudan	Facility-based	Retrospective review	Postnatal mother	Chart review	15642	328	2.1	**Neonatal**: Prematurity 50(22.9%) Still birth 37(15.8), Asphyxia 62(41.3%), Prematurity 57 (38%), 2 congenital malformation **Maternal:** Anemia 12 (13%) APH 1(1.1%), PROM 5 (5.4%) Polyhydraminious 1(1.1%) Preeclampsia 65 (70.7%)
Gessessew,A.,2007 [38]	Ethiopia	Facility-based	Descriptive retrospective study	Postnatal mother	Chart review	7226	99	1.37	**Neonatal:** Malpresentation (14.1%) **Maternal:** Preterm labor (39.4%), PROM (31.3%), APH (11.1%), PPH (9.1%), PE (9.1%), Maternal death (3%),
Habib,et al, 2008 [39]	Tanzania	Facility-based	Comparative cross-sectional	Postnatal mother	Chart review	15255	771	5.05	**Maternal**: cesarean section (40%)
Dafallah SE, Yousif EM,2004 [30]	Sudan	Facility-based	Cohort study	Pregnant mother	Observation and interview	44605	597	1.34	**Neonatal**: Perinatal mortality rate was 115/1000 for twin **Maternal** : MMR=was 35.8/100.000 Pre-term labor, 35.5%; Cesarean section 53.1%
Abebaw et al., 2021 [40]	Ethiopia	Facility based	Cross-sectional	Newborn	Chart review	24561	748	3.05	**Neonata**l: LBW (9.1%), Stillbirth (42%), APGAR Score <7 (9.1%), Prematurity (66%), Prenatal death (23%), **Maternal:** HDP (10.6%), PROM (8.8%)
Moller B. et al., 1989 [29]	Tanzania	Community based	Cross-sectional	Pregnant women	Interview	719	49	6.8	**Neonatal:** perinatal mortality (23%), Preterm (16%)
Chiwanga, E.S et al,. 2014 [41]	Tanzania	DHS	Cross-sectional	Pregnant Mother	Chart review	33997	822	2.1	**Maternal:** Preterm labor (9.4%) MMR (0.07%), Preeclampsia (9.4%), APH (7.2%) Anemia (2.0%) C/s (42.6%)
Gebremedhin, S. (2015) [42]	Burundi	DHS	cross-sectional	postnatal mothers	DHS chart review	4267	55	1.3	Neonatal mortality (5.5%)
Temesgen,T., 2015 [43]	Ethiopia	Facility-based	Case-control	Pregnant mother	Interview	3812	144	3.7	Not Applicable (N/A)
Gebremedhin, S. (2015) [42]	Comoros	DHS	cross-sectional	postnatal mothers	DHS chart review	3149	66	2.1	N/A
Gebremedhin, S. (2015) [42]	Ethiopia	DHS	cross-sectional	postnatal mothers	DHS chart review	11654	136	1.2	N/A
Gebremedhin, S. (2015) [42]	Kenya	DHS	Cross-sectional	postnatal mothers	DHS chart review	6079	81	1.3	N/A
Gebremedhin, S. (2015) [42]	Rwanda	DHS	Cross-section	postnatal mothers	DHS chart review	9002	134	1.5	N/A
Gebremedhin, S. (2015) [42]	Tanzania	DHS	Cross-section	postnatal mothers	DHS chart review	8648	146	1.7	N/A
Gebremedhin, S. (2015) [42]	Uganda	DHS	Cross-section	postnatal mothers	DHS chart review	7878	129	1.6	N/A
Sikosana, ML, 2006 [44]	Tanzania	Facility-based	Case- control	Postnatal mother	Delivery chart review	1922	57	2.9	N/A
MWITA S et al., 2022 [45]	Tanzania	Facility based	Retrospective cohort	Pregnant women	chart review	1012	210	19.6	N/A
Guo, G. and Grummer-Strawn, L.M., 1993 [46]	Burundi	DHS	Cross-sectional	Pregnant women	Interview	3811	66	1.7	N/A
Guo, G. and Grummer-Strawn, L.M., 1993 [46]	Kenya	DHS	Cross-sectional	Pregnant women	Interview	6985	196	2.8	N/A
Guo, G. and Grummer-Strawn, L.M., 1993 [46]	Uganda	DHS	Cross-sectional	Pregnant women	Interview	4959	162	3.3	N/A
Justesen, A. and Kunst, A., 2000 [47]	Tanzania	DHS	Cross-sectional	Newborns	secondary data analysis	8132	280	3.4	N/A
Bellizzi, S., et al, 2018 [48]	Burundi	DHS	Cross-sectional	Postnatal women	Chart review	7405	168	2.3	N/A
Bellizzi, S., et al, 2018 [48]	Comoros	DHS	Cross-sectional	Postnatal women	Chart review	3082	126	4.1	N/A
Bellizzi, S., et al, 2018 [48]	Ethiopia	DHS	Cross-sectional	Postnatal women	Chart review	11166	314	2.8	N/A
Bellizzi, S., et al, 2018 [48]	Kenya	DHS	Cross-sectional	Postnatal women	Chart review	5862	168	2.9	N/A
Bellizzi, S., et al, 2018 [48]	Rwanda	DHS	Cross-sectional	Postnatal women	Chart review	8678	246	2.8	N/A
Bellizzi, S., et al, 2018 [48]	Tanzania	DHS	Cross-sectional	Postnatal women	Chart review	7705	210	2.7	N/A
Bellizzi, S., et al, 2018 [48]	Uganda	DHS	Cross-sectional	Postnatal women	Chart review	7535	226	3	N/A

### Prevalence of twin pregnancy in Eastern Africa

**Fig. 2:**
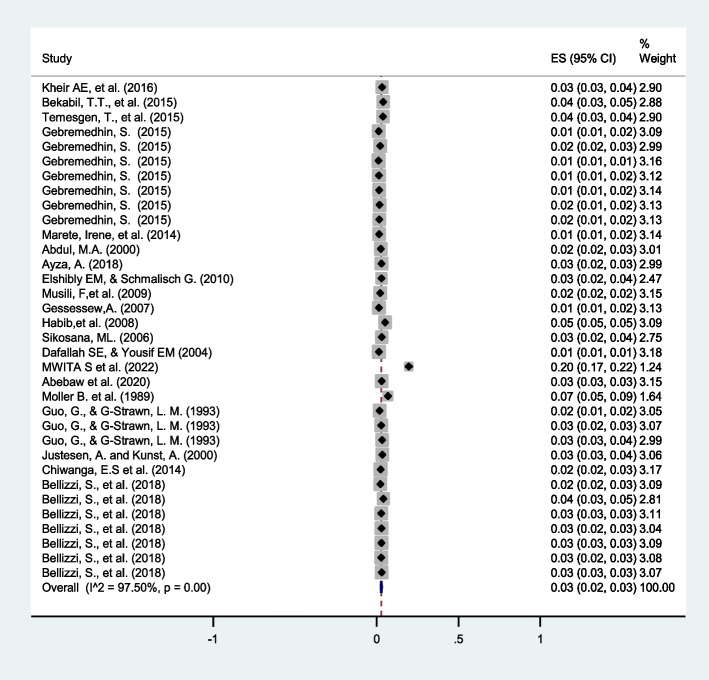
Forest plot showing the pooled prevalence of twin pregnancy in Eastern Africa

**Fig. 3:**
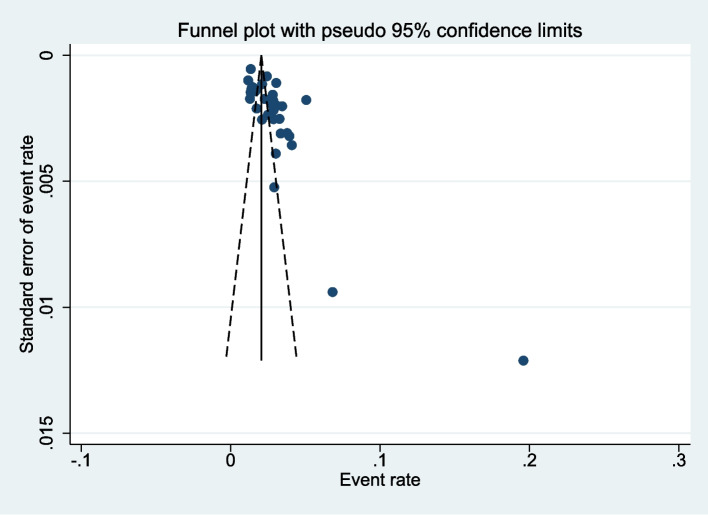
Funnel plot meta-analysis of twin pregnancy prevalence in Eastern Africa

The prevalence of twin pregnancy in eastern Africa varied from 1.2% [42] to 19.6% [45]. The random-effects model analysis from identified 34 studies revealed that the overall pooled prevalence of twin pregnancy in eastern Africa was 3% (95%CI: 2– 3) with high heterogeneity observed across the included studies (I^2^ = 97.5%, *p* = < 0.001) (Fig. 2). The funnel plot was asymmetric (Fig. 3).

### Subgroup analysis

**Fig. 4:**
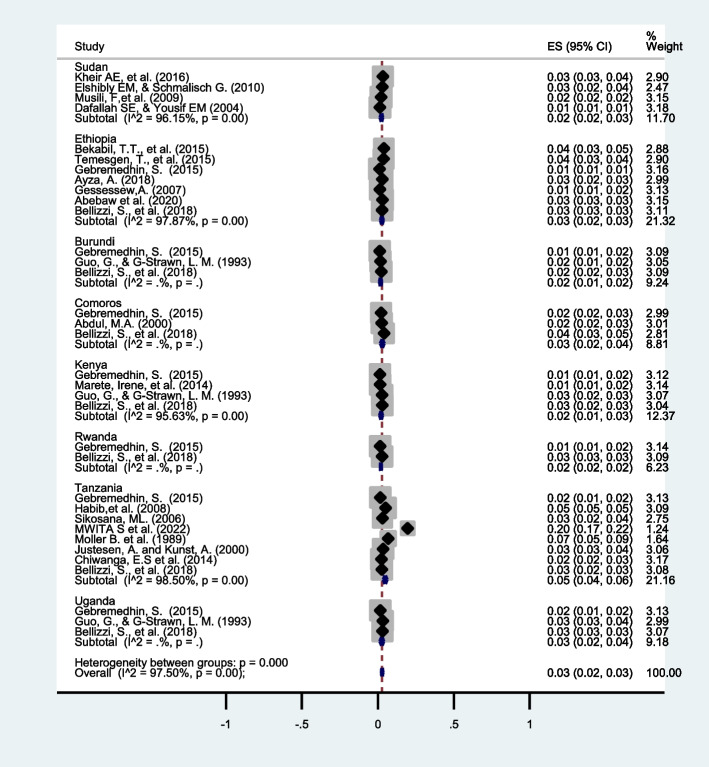
Country-based subgroup analysis of twin pregnancy in Eastern Africa

In this meta-analysis, the prevalence of twin pregnancy in eastern Africa has been computed and subgroup analysis by year and country were conducted. Countries-based subgroup analysis revealed that the highest prevalence of twin pregnancy in Tanzania (5%, 95% CI: 4-6) with I^2^ = 98.50% and a *p*-value < 0.001 while the lowest prevalence was observed in Sudan, Burundi, Kenya, and Rwanda. However, the subgroup analysis computed by the year of the study showed no evidence of heterogeneity (Fig. 4).

### Adverse perinatal outcomes of twin pregnancy in Eastern Africa

#### Adverse neonatal outcomes

##### Low birth weight:

**Fig. 5:**
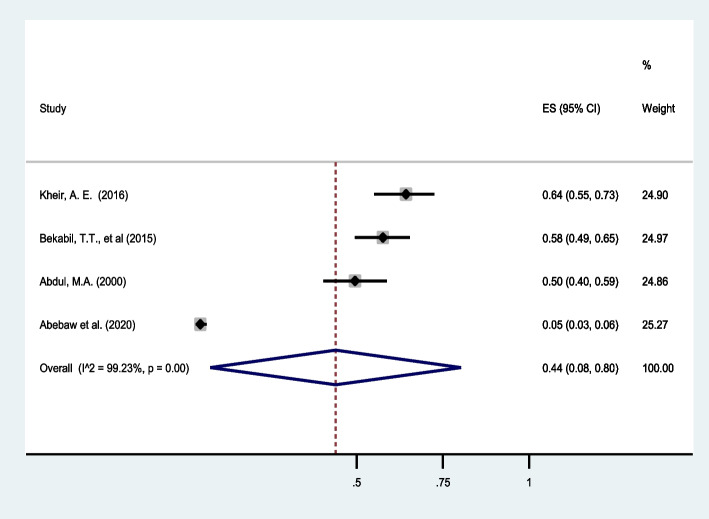
Forest plot showing the pooled prevalence of low birth weight among twin pregnancies in eastern Africa

Low birth weight (<2500gm) in twin pregnancies was reported in four studies conducted in eastern Africa [31, 32, 34, 40]. The pooled estimate indicated that nearly half of the twin pregnancies result in low birth weight (44%, 95%CI: 8 to 80) with high heterogeneity (I^2^ = 99.23%) in Eastern Africa (Fig. 5).

##### Neonatal mortality:

**Fig. 6:**
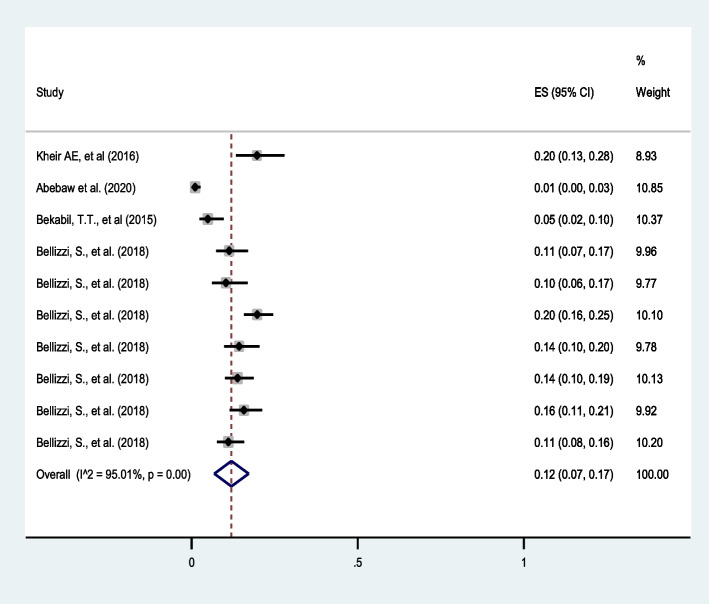
Forest plot showing the pooled prevalence of neonatal mortality among twin pregnancies in eastern Africa

Ten studies [31, 32, 40, 48] reported neonatal mortality in twin pregnancies and the pooled estimate revealed that one in ten twin pregnancies results in neonatal mortality (12%: 95%CI: 7 to 17) with I^2^ = 95.01%.The highest rate was observed in Sudan [31] and the lowest was in Ethiopia [40] (Fig. 6).

##### Prematurity:

**Fig. 7:**
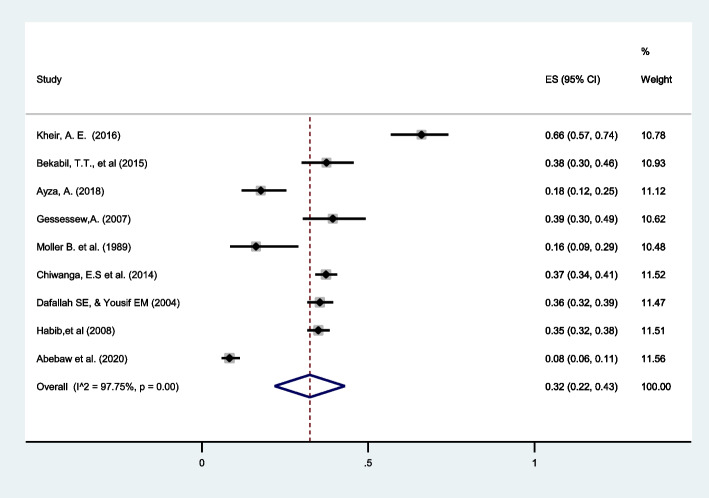
Forest plot showing the pooled prevalence of prematurity among twin pregnancies in eastern Africa

Nnine articles reported on prematurity in twin pregnancies revealing pooled prevalence of 32% (95%CI: 22 to 43) with high heterogeneity (I^2^ = 97.75). The prevalence varied from 8% in Ethiopia to 66% in Sudan (Fig. 7).

##### Stillbirth:

**Fig. 8:**
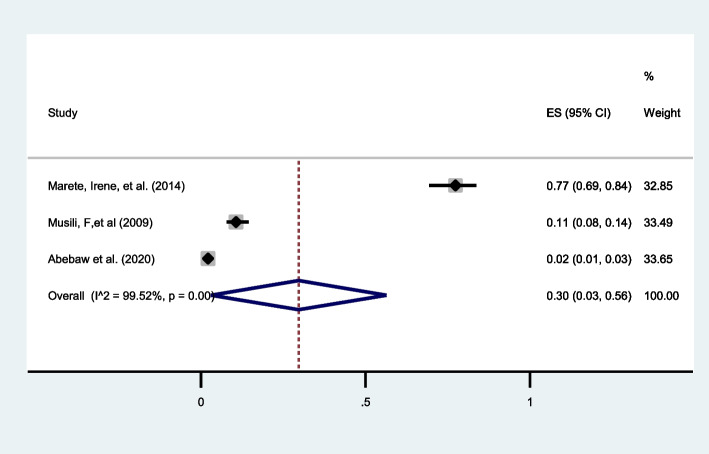
Forest plot showing the pooled prevalence of stillbirth among twin pregnancies in eastern Africa

From the pooled prevalence of the three studies [33, 37, 40] reporting the incidence of stillbirth in twin pregnancies, it was found that one in three twin pregnancies (30%; 95%CI: 3 to 56) were complicated with stillbirth with I^2^ = 99.52% (Fig. 8).

##### Perinatal mortality:

**Fig. 9:**
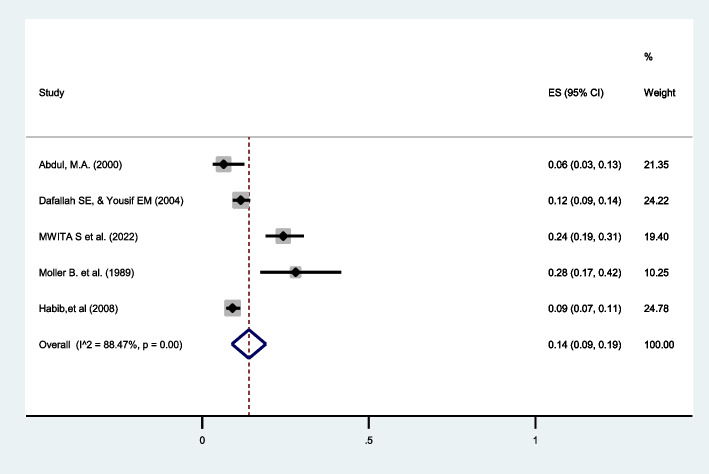
Forest plot showing the pooled prevalence of perinatal mortality among twin pregnancies in eastern Africa

Perinatal mortality in twin pregnancies was reported in five studies [29, 30, 34, 39, 45]. The review identified the pooled prevalence of perinatal mortality was 14% (95%CI: 9 to 19) with I^2^ = 88.47% (Fig. 9).

##### Low APGAR score:

**Fig. 10:**
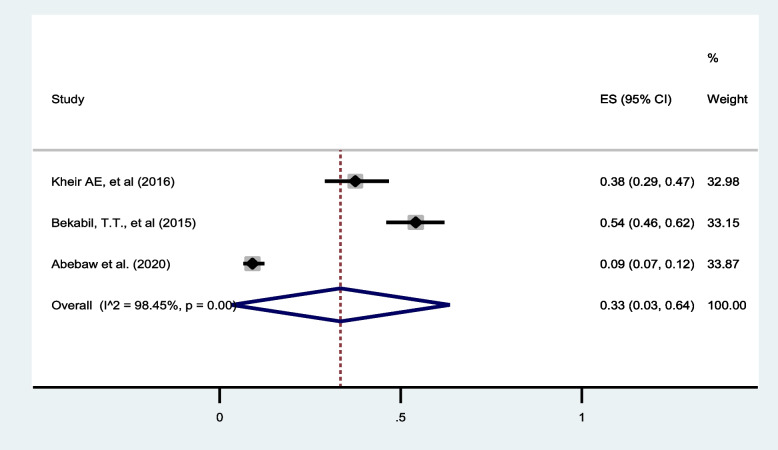
Forest plot showing the pooled prevalence of low APGAR score among twin pregnancies in eastern Africa

The review identified three studies [31, 32, 40] reporting the presence of low APGAR score (<7) in twin pregnancies. The pooled estimate indicated a prevalence of 33% (95% CI: 3 to 64) with I^2^ = 98.45% (Fig. 10).

##### Admission to the neonatal intensive care unit (NICU):

**Fig. 11:**
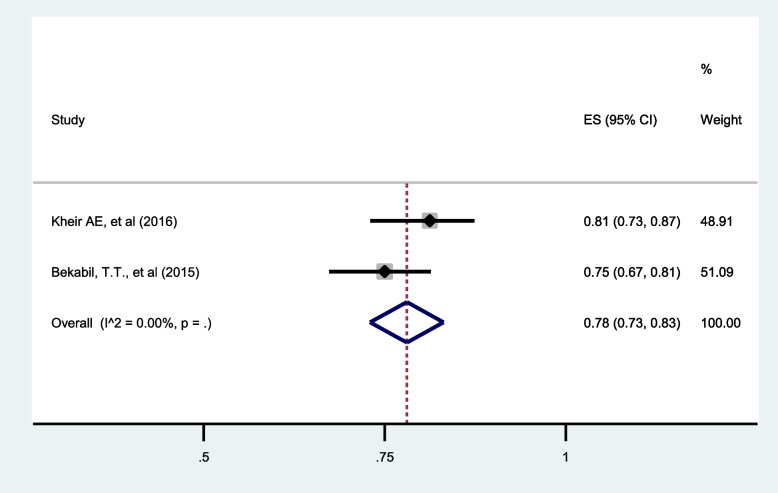
Forest plot showing the pooled prevalence of NICUadmission among twin pregnancies in eastern Africa

Data from two studies [31, 32] reporting NICU admission in twin pregnancy revealed that 78% (95%CI: 73 to 83) of neonates among twin pregnancies were admitted to NICU (Fig. 11).

### Adverse maternal outcomes

#### Hypertensive disorder in pregnancy (HDP)

**Fig. 12:**
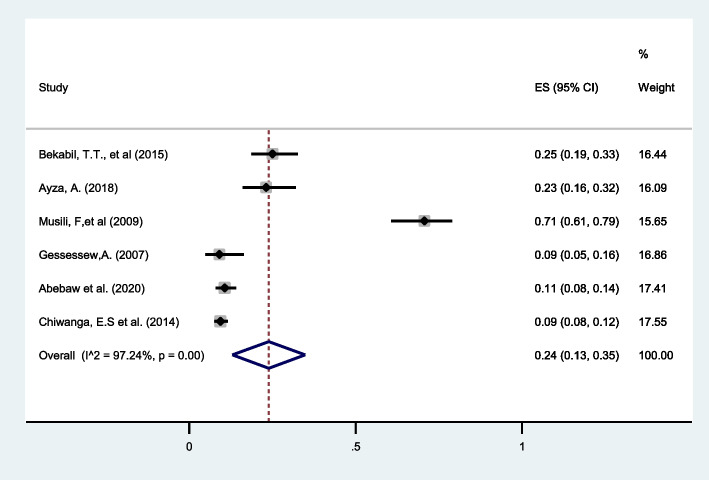
Forest plot showing the pooled prevalence of HDP among twin pregnancies in eastern Africa

Six studies reported maternal complication of HDP in twin pregnancy [32, 35, 37, 38, 40, 41]. The review found that more than a quarter of twin pregnancies were complicated with HDP (25%, 95%CI: 13 to 35) with (I^2^ = 97.24%) and the highest percentage (71%) of this complication was reported from Kenya (Fig. 12).

#### Postpartum Hemorrhage (PPH)

**Fig. 13:**
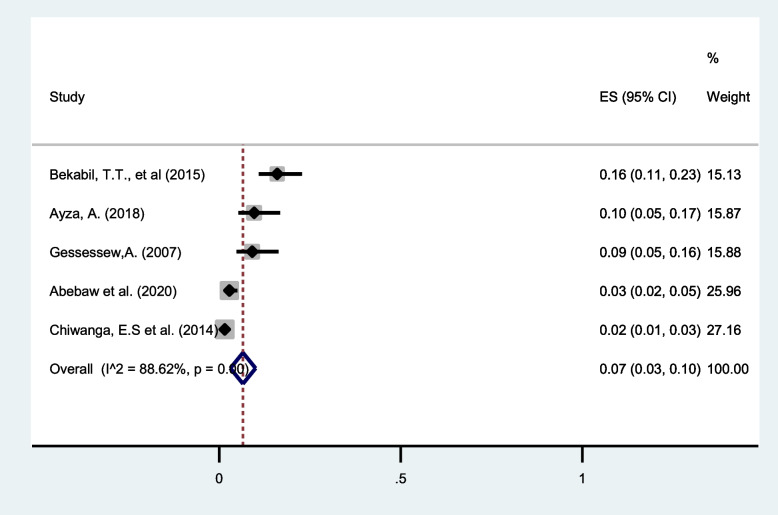
Forest plot showing the pooled prevalence of PPH among twin pregnancies in eastern Africa

PPH as a maternal complication in twin pregnancy was reported in five different studies [32, 35, 38, 40, 41]. The pooled prevalence of this meta-analysis estimate shows a significant number of twin pregnancies were complicated with PPH (7%, 95%CI: 3 to 10) with I^2^ = 88.62% (Fig. 13).

#### Caesarean section (C/S)

**Fig. 14:**
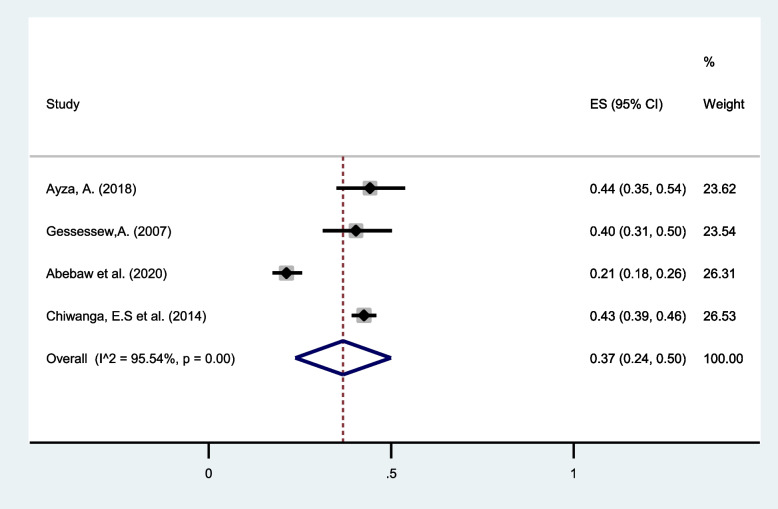
Forest plot showing a pooled prevalence of cesarean section among twin pregnancies in eastern Africa

C/S as a maternal complication of twin pregnancy was reported in four different studies [35, 38, 40, 41] conducted in eastern Africa. Pooled estimates show that one-third of twin pregnancies undergo C/S (37%, 95% CI: 24 to 50) with I^2^ = 95.24% (Fig. 14).

#### Premature rupture of membrane (PROM)

**Fig. 15:**
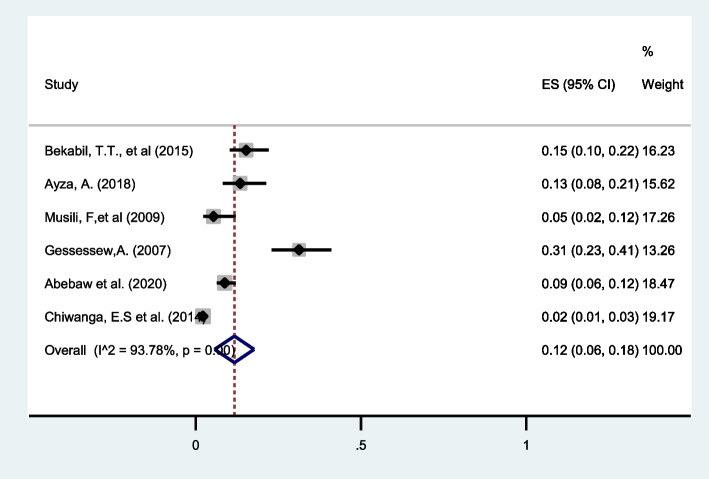
Forest plot showing the pooled prevalence of PROM among twin pregnancies in eastern Africa

Six studies from eastern Africa reported PROM as a maternal complication in twin pregnancies. More than a tenth (12%, 95%CI: 6 to 18) of twin pregnancies developed PROM as complications of pregnancy with I^2^ = 93.78% (Fig. 15).

#### Maternal mortality

**Fig. 16:**
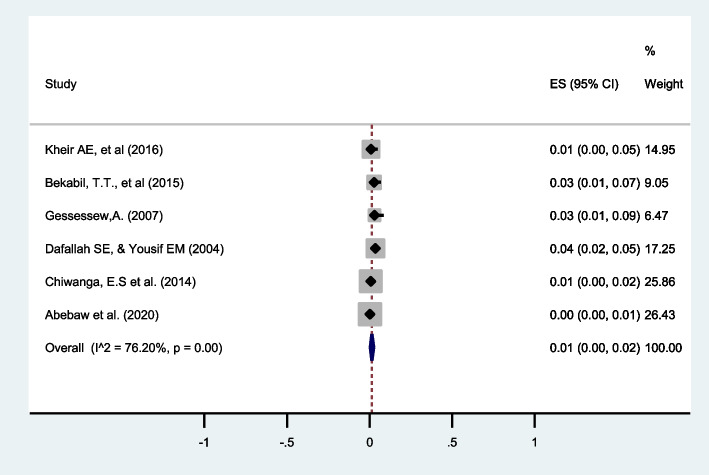
Forest plot showing the pooled prevalence of maternal mortality among twin pregnancies in eastern Africa

The risk of maternal mortality in twin pregnancy was reported by six studies [30–32, 38, 40, 41]. The pooled estimate from these six studies revealed that a significant number of pregnant mothers (1%, 95%CI: 0 to 1) died following twin pregnancies in eastern Africa with I^2^ = 76.20% (Fig. 16).

#### Meta-regression

Meta-regression was performed to examine the impact of publication year and sample size on heterogeneity revealing no heterogeneity among the studies based on these factors (Table 2).

**Table 2:** Meta-regression analysis to check heterogeneity on twin pregnancy in Eastern Africa, 2023

Variables	Coefficients	SE	P	[95% Conf. Interval]
Publication year	0. 0002555	0. 0005668	0.655	-0.0009022, 0.0014131
Sample size	-9.50e-07	5.61e-07	0.101	-2.10e-06, 1.95e-07
Setting	-0.0161392	.0102543	0.126	-.0370812, 0.0048028

## Discussion

This comprehensive study offers valuable insight into perinatal outcomes among twin pregnancies in Eastern Africa. Twin pregnancies pose risks during pregnancy, labour, and delivery as well as during the postnatal period manifesting as preterm delivery, antepartum haemorrhage, postpartum hemorrhage and twin-related complications.

The pooled prevalence of twin pregnancy in eastern Africa was (3%, 95% CI: 2– 3). This study finding aligns with a studies conducted among 23 low and middle-income countries [14], the United States [49, 50], Botswana [51], different countries [52], developing world [53], and developed world [54]. The consistency in prevalence across these studies suggests stability despite sociocultural variations. It’s worth noting that the increasing rate of twin pregnancies in recent times is attributed to technological advancement and infertility treatment.

Adverse perinatal outcomes are more common among twin pregnancies as evidenced by our study pooled results. Approximately 32% of twin pregnancies experienced preterm delivery due to various complications the increased likelihood of spontaneous preterm labor [55, 56]. Furthermore 44% of twin pregnancies resulted in low birthweight. This could be evidenced by twin pregnancies’ increased demand for nutrients and oxygenated blood [57]. Adverse outcomes of twin pregnancy such as low APGAR score (33%) and NICU admission (78%) were also reported. This is likely due to the majority of twin pregnancies being born preterm and with low birth weight, resulting in low APGAR scores and necessitating NICU admission. A study conducting in Netherlands supports these finding [58].

In addition our study identified perinatal mortality (14%), stillbirth (30%), and neonatal mortality (12%) among twin pregnancies in Eastern Africa. This high mortality may be attributed to immaturity and twin-related factors as suggested by previous studies [59]. Increased perinatal and obstetric complications among twins could be another contributing factors to the elevated mortality rates [60, 61].

Twin pregnancy pose various complications to the mother as well. Our study revealed that nearly one-fourth (25%) of twin pregnancies develop hypertensive disorder of pregnancy (HDP) in Eastern Africa. The occurrence of HDP is proportionate to the number of fetuses, given its pathophysiology related to placental mass, which is higher with twin pregnancies [62, 63]. Additionally, maternal complication like PPH (7%) and PROM (12%) was reported among twin pregnancy. This may be attributed to the over-distention of the uterus from twin pregnancy serving as as a mechanical cause of PPH [4]. Cesarean section was reported in approximately 37% of twin pregnancies, likely justified by high perinatal and intrapartum complications among twins making C/S a life-saving intervention [19, 64]. Additionally, 34 (1%) cases of maternal mortality was reported among twins in Eastern Africa, possibly linked to increased risk of morbidity associated with multiple pregnancies [21, 61].

Generally, a complication from a twin pregnancy differ from a singleton pregnancies, introducing secondary complications. The unique challenges of twin pregnancies such as the need of special antenatal care and prolonged hospital admission due to preterm delivery, contribute to increased health costs and affect quality of life. Also increased prevalence of severe handicaps and cerebral palsy among twins negatively impact the quality of life [65]. Given the high risk of complication associated with twin pregnancy, it is imperative for countries to develop targeted strategies aimed at reducing adverse pregnancy outcomes in multiple pregnancies.

### Implication of the study

The study provide compressive overview of the prevalence of twin pregnancies in Eastern Africa by synthesizing data from multiple studies. The information is crucial to tailor intervention and improve maternal and neonatal outcome. This information can inform clinical guideline for the management of twin pregnancies. This study contribute significantly to the body of knowledge, influencing clinical practice, policy development and future research endeavor in the region.

## Conclusion

The burden of twin pregnancy and its adverse outcome need attention. Adverse neonatal outcomes like NICU admission, low birth weight, low APGAR score, prematurity, stillbirth, neonatal mortality and maternal complications like HDP, PPH, cesarean section, PROM and maternal mortality are more common among twin pregnancies than single-tone pregnancies. Special care for mothers with twin pregnancies is recommended to mitigate adverse pregnancy outcomes.

### Strength and limitation

The study provides compressive overview of twin pregnancy and its adverse outcome in Eastern Africa, offering a more representative perspective than a single study. However, caution’s is warranted in interpreting the finding due to considerable heterogeneity across the included studies.

### Supplementary Information

**Additional file 1.** PRISMA checklist.

**Additional file 2.** Searching strategy.

**Additional file 3.** Appraisal.

## Data Availability

Additional data can be available from the corresponding author upon reasonable request.
