# Supplementary material for: Prevalence and adverse outcomes of twin pregnancy in Eastern Africa: a systematic review and meta-analysis
Source: BMC Pregnancy Childbirth. 2024 Feb 29;24:169. doi: 10.1186/s12884-024-06326-0 (PMC10905881; doi:10.1186/s12884-024-06326-0)
Supplement: Supplementary file 2 — Additional file 2. Searching strategy. [file 12884_2024_6326_MOESM2_ESM.docx]

**Additional file 2:** Searching strategy for the prevalence of twin pregnancy and/or adverse maternal and fetal outcome in eastern Africa: a systematic review and meta-analysis

1. **Sample search string for CINHAL database, EBSCOhost Interface**

| **#** | **Query** | **Limiters/Expanders** | **Last Run Via** | **Results** |
| --- | --- | --- | --- | --- |
| **S8** | ((eastern Africa) AND (S1 OR S2 OR S3 OR S4 OR S5)) AND (S6 OR S7) | Expanders - Also search within the full text of the articles  Search modes - Boolean/Phrase | Interface - EBSCOhost Research Databases  Search Screen - Advanced Search  Database - CINAHL | **1** |
| **S7** | (eastern Africa) AND (S1 OR S2 OR S3 OR S4 OR S5) | Expanders - Also search within the full text of the articles  Search modes - Boolean/Phrase | Interface - EBSCOhost Research Databases  Search Screen - Advanced Search  Database - CINAHL | 1 |
| S6 | Eastern Africa | Expanders - Also search within the full text of the articles  Search modes - Boolean/Phrase | Interface - EBSCOhost Research Databases  Search Screen - Advanced Search  Database - CINAHL | 169 |
| S5 | Multiple pregnancies | Expanders - Also search within the full text of the articles  Search modes - Boolean/Phrase | Interface - EBSCOhost Research Databases  Search Screen - Advanced Search  Database - CINAHL | 2328 |
| S4 | Multiple gestation | Expanders - Also search within the full text of the articles  Search modes - Boolean/Phrase | Interface - EBSCOhost Research Databases  Search Screen - Advanced Search  Database - CINAHL | 818 |
| S3 | Twins pregnancy | Expanders - Also search within the full text of the articles  Search modes - Boolean/Phrase | Interface - EBSCOhost Research Databases  Search Screen - Advanced Search  Database - CINAHL | 2657 |
| S2 | Twin pregnancies | Expanders - Also search within the full text of the articles  Search modes - Boolean/Phrase | Interface - EBSCOhost Research Databases  Search Screen - Advanced Search  Database - CINAHL | 2960 |
| S1 | Twin pregnancy | \|  \| Expanders - Also search within the full text of the articles  Search modes - Boolean/Phrase \| \| --- \| --- \| | Interface - EBSCOhost Research Databases  Search Screen - Advanced Search  Database - CINAHL | 2960 |

1. **For other databases**

| Databases | Searching terms | Number of studies |
| --- | --- | --- |
| PubMed | ("pregnancy, twin"[MeSH Terms] OR ("pregnancy, twin"[MeSH Terms] OR ("pregnancy"[All Fields] AND "twin"[All Fields]) OR "twin pregnancy"[All Fields] OR ("twin"[All Fields] AND "pregnancies"[All Fields]) OR "twin pregnancies"[All Fields]) OR ("pregnancy, twin"[MeSH Terms] OR ("pregnancy"[All Fields] AND "twin"[All Fields]) OR "twin pregnancy"[All Fields] OR ("twins"[All Fields] AND "pregnancy"[All Fields]) OR "twins pregnancy"[All Fields]) OR ("pregnancy, multiple"[MeSH Terms] OR ("pregnancy"[All Fields] AND "multiple"[All Fields]) OR "multiple pregnancy"[All Fields] OR ("multiple"[All Fields] AND "gestation"[All Fields]) OR "multiple gestation"[All Fields]) OR "pregnancy, multiple"[MeSH Terms]) AND "africa, eastern"[MeSH Terms] | 740 |
| Google scholar | Prevalence of twin pregnancy and/or adverse maternal and fetal outcome in eastern Africa | 677 |
| Scopus | (twin AND pregnancy) OR ( twin AND pregnancies ) OR ( twins AND pregnancy ) OR ( multiple AND gestation ) OR ( multiple AND pregnancy ) AND (perinatal OR maternal OR fetal outcomes) AND (eastern AND africa ) | 490 |
| Web of Science | (twin AND pregnancy) OR ( twin AND pregnancies ) OR ( twins AND pregnancy ) OR ( multiple AND gestation ) OR ( multiple AND pregnancy ) AND (perinatal OR maternal OR fetal outcomes) AND (eastern AND africa ) | 180 |
| Others databases |  | 30 |
| Total retrieved |  | 2118 |
| Included |  | 34 |
