# Supplementary material for: Prevalence and adverse outcomes of twin pregnancy in Eastern Africa: a systematic review and meta-analysis
Source: BMC Pregnancy Childbirth. 2024 Feb 29;24:169. doi: 10.1186/s12884-024-06326-0 (PMC10905881; doi:10.1186/s12884-024-06326-0)
Supplement: Supplementary file 3 — Additional file 3. Appraisal. [file 12884_2024_6326_MOESM3_ESM.docx]

**Appraisal**

Critical appraisal check list of quantitative studies of maternal and neonatal outcome of twin pregnancy in Eastern Africa. (1 =yes, 0=no/not mentioned); total score=8

| Studies | Q1 | Q2 | Q3 | Q4 | Q5 | Q6 | Q7 | Q8 | Total score | Remark |
| --- | --- | --- | --- | --- | --- | --- | --- | --- | --- | --- |
| Kheir, A. E., et al | Y | Y | Y | Y | Y | Y | Y | Y | 8/8 |  |
| Bekabil, T.T., et al | Y | Y | Y | Y | Y | Y | Y | Y | 8/8 |  |
| Temesgen, T | Y | Y | Y | Y | Y | Y | Y | Y | 8/8 |  |
| Gebremedhin, S. | Y | Y | Y | Y | U | Y | Y | Y | 7/8 | The study focus on risk of twin pregnancy rather than maternal and neonatal outcome of twin pregnancy. |
| Marete, Irene, et al. | Y | Y | Y | N | Y | Y | Y | Y | 7/8 | Result was pooled except for prevalence of twin pregnancy as study include six country |
| Abdul, M.A | N | N | Y | Y | Y | Y | Y | Y | 6/8 |  |
| Ayza, A | Y | Y | Y | Y | Y | Y | Y | Y | 8/8 |  |
| Elshibly EM, Schmalisch G | Y | N | Y | Y | Y | Y | Y | Y | 7/8 |  |
| Musili, F,et al | Y | Y | Y | Y | Y | U | Y | Y | 7/8 |  |
| Gessessew, A | Y | N | Y | U | Y | Y | Y | Y | 6/8 |  |
| Habib,et al | Y | Y | Y | Y | Y | Y | Y | Y | 8/8 |  |
| Sikosana, ML | U | N | Y | Y | Y | Y | Y | Y | 6/8 |  |
| Dafallah SE, Yousif EM | N | Y | Y | Y | Y | Y | Y | Y | 7/8 |  |
| MWITA S et al., | Y | Y | Y | Y | Y | N | U | Y | 6/8 | The study Include only preterm pregnancy that had high chance to develop complication. Additional total delivery during study period was not given which may result in increase the proportion of twin pregnancy. The study focus on effect of antenatal corticosteroid exposure on pregnancy outcome, rather than outcome of twin pregnancy. |
| Abebaw et al | Y | Y | Y | Y | Y | Y | Y | Y | 8/8 |  |
| Moller B. et al | Y | Y | Y | N | Y | U | Y | Y | 6/8 |  |
| Guo, G. and Grummer-Strawn, L.M | Y | N | Y | Y | Y | Y | Y | Y | 7/8 |  |
| Justesen, A. and Kunst, A | Y | Y | Y | Y | Y | Y | Y | Y | 8/8 |  |
| Chiwanga, E.S et al | Y | Y | Y | Y | Y | Y | Y | Y | 8/8 |  |
| Bellizzi, S., et al | Y | Y | Y | Y | Y | Y | Y | Y | 8/8 |  |

Notes:

Q1 - Were the criteria for inclusion in the sample clearly defined?

Q2 - Were the study subjects and the setting described in detail?

Q3 - Was the exposure measured in a valid and reliable way?

Q4 - Were objective, standard criteria used for measurement of the condition?

Q5 - Were confounding factors identified?

Q6 - Were strategies to deal with confounding factors stated?

Q7 - Were the outcomes measured in a valid and reliable way?

Q8 - Was appropriate statistical analysis used?

Abbreviations: Y, yes; N, no; U, unclear.
